# Associations between maternal gestational diabetes metformin or insulin treatment and offspring growth trajectories from birth to 60 months of age: Findings from the Born in Bradford (BiB) study

**DOI:** 10.1111/dme.15204

**Published:** 2023-08-26

**Authors:** Gilberte Martine‐Edith, William Johnson, Emily S. Petherick

**Affiliations:** ^1^ School of Sport, Exercise and Health Sciences Loughborough University Loughborough UK

**Keywords:** Born in Bradford, gestational diabetes, growth trajectories, insulin, metformin

## Abstract

**Aim:**

To investigate the associations between gestational diabetes mellitus (GDM) metformin or insulin treatment and offspring growth trajectories from 0 to 60 months.

**Methods:**

Participants were from the Born in Bradford birth cohort study. Using covariate‐adjusted multilevel linear spline models (4 splines: 0–1.6, 1.6–6, 6–17 and 17–60 months), we compared weight, height and body mass index (BMI) z‐score trajectories of: (1) 76 offspring exposed to metformin (OGDM‐Metformin) and 420 offspring exposed to insulin (OGDM‐Insulin); (2) OGDM‐Metformin and 9171 offspring not exposed to GDM (No‐GDM); (3) OGDM‐Insulin and No‐GDM.

**Results:**

(1) OGDM‐Metformin had comparable growth trajectories to OGDM‐Insulin from 0 to 60 months. (2) OGDM‐Metformin had a lower mean birthweight z‐score than No‐GDM. OGDM‐Metformin had faster changes in height z‐score (0.13 [95% CI 0.026, 0.24]) from 17 to 60 months and by 60 months, had comparable mean BMI z‐score to No‐GDM. (3) OGDM‐insulin had lower mean birthweight and height z‐scores than No‐GDM. OGDM‐Insulin had faster changes in weight (0.32 [0.021, 0.62]) and height (0.50 [0.087, 0.91]) from 1.6 to 6 months and by 60 months, had comparable mean BMI z‐score to No‐GDM.

**Conclusions:**

GDM metformin treatment was not associated with differences in offspring growth trajectories compared to insulin treatment. Both metformin and insulin‐exposed offspring had comparable BMI z‐score to No‐GDM by 60 months.

**What's new?**

- Metformin, unlike insulin, crosses the placenta and was shown to reduce placental energy production which may impact on offspring growth but no studies had longitudinally compared the growth trajectories of metformin‐ and insulin‐exposed offspring.
- Metformin‐exposed offspring had comparable growth trajectories to insulin‐exposed offspring from 0 to 60 months of age.
- By 60 months of age, both metformin‐ and insulin‐exposed offspring had comparable mean BMI z‐score to offspring not exposed to GDM.
- There is no indication that using metformin as an alternative to insulin for GDM treatment is associated with differences in offspring growth trajectories from 0 to 60 months of age.

## 1 INTRODUCTION

Gestational diabetes mellitus (GDM) is a common complication of pregnancy, characterised by an impaired tolerance to glucose.^1^ In 2021, the global prevalence of GDM was 14.2% according to the International Diabetes Federation.^2^ In the United Kingdom (UK), it is estimated that 30,625 births each year or 4.4% of births are affected by GDM.^3^

Insulin has historically been the first‐line pharmaceutical agent for GDM if initial lifestyle changes advice (including dietary and exercise advice) are insufficient to restore euglycaemia.^3^ Since the Metformin in Gestational diabetes (MiG) trial, metformin, another pharmaceutical agent, has been increasingly recommended as an alternative or supplement to insulin in GDM treatment.^3^, ^4^ Despite this transition in GDM treatment recommendations, there are uncertainties around metformin safety in pregnancy. Metformin, unlike insulin, crosses the placenta and recent evidence showed that, in human cells, it reduces placental energy production, an energy that is essential in maintaining adequate fetal growth.^5^ Further, research demonstrated that metformin‐exposed offspring had lower birthweight^6^ but higher weight and body mass index (BMI) than insulin‐exposed offspring in childhood.^7^, ^8^ This may indicate that metformin‐exposed children experience faster post‐natal growth^6^ however, it is unclear whether this is consistent across different growth periods as no studies to date directly assessed their growth trajectories longitudinally, using repeated growth measures over time. Given the associations between faster post‐natal growth and greater obesity risk in childhood,^9^ it is essential to investigate the growth trajectories of metformin‐exposed children to determine whether they differ from those of insulin‐exposed children, according to growth period. It is also of clinical importance to compare metformin and insulin‐exposed offspring to offspring not exposed to GDM to determine whether metformin or insulin treatment is associated with a change in the associations previously observed between GDM exposure and higher obesity and adiposity risk in childhood.^10^

Using data from the UK Born in Bradford (BiB) cohort, we compared the weight, height and BMI z‐score trajectories from 0 to 60 months of (1) offspring exposed to metformin and those exposed to insulin and (2) offspring exposed to metformin or insulin and those not exposed to GDM.

## 2 PARTICIPANT AND METHODS

### 2.1 Study

The longitudinal prospective Born in Bradford (BiB) birth cohort study was conducted at the Bradford Royal Infirmary between 2007 and 2010, recruiting 12,453 women and 13,858 children. As one of the UK's largest cities, Bradford is located in the north of England and has a population of more than 500,000 individuals.^11^

All women booked for delivery at the Bradford Royal Infirmary were offered GDM screening using the 2 h 75 g oral glucose tolerance test (OGTT) at 26–28 weeks of pregnancy. GDM diagnosis was made according to the modified 1999 WHO criteria (fasting glucose concentration ≥6.1 mmol/L or 2‐h post‐load glucose ≥7.8 mmol/L).^12^ If a woman was diagnosed with GDM, changes in dietary habits along with a minimum of 30‐min daily walks were recommended by the clinical team. If lifestyle changes were sufficient to achieve glucose targets (fasting plasma glucose: 4.0–5.5 mmol/L; 2‐h postprandial: <7.5 mmol/L), it was advised for those changes to be continued without additional pharmaceutical treatment. If lifestyle changes were insufficient, insulin injections were prescribed in the first part of the study (04/2007‐03/2009). From April 2009, either insulin injections or metformin tablets (850 mg/L, twice a day) were prescribed if lifestyle changes were insufficient to restore euglycaemia. Ethical approval for the study was provided by Bradford Research Ethics Committee (Ref 07/H1302/112). All participants provided written consent for the BiB study.

### 2.2 Sample

**FIGURE 1:**
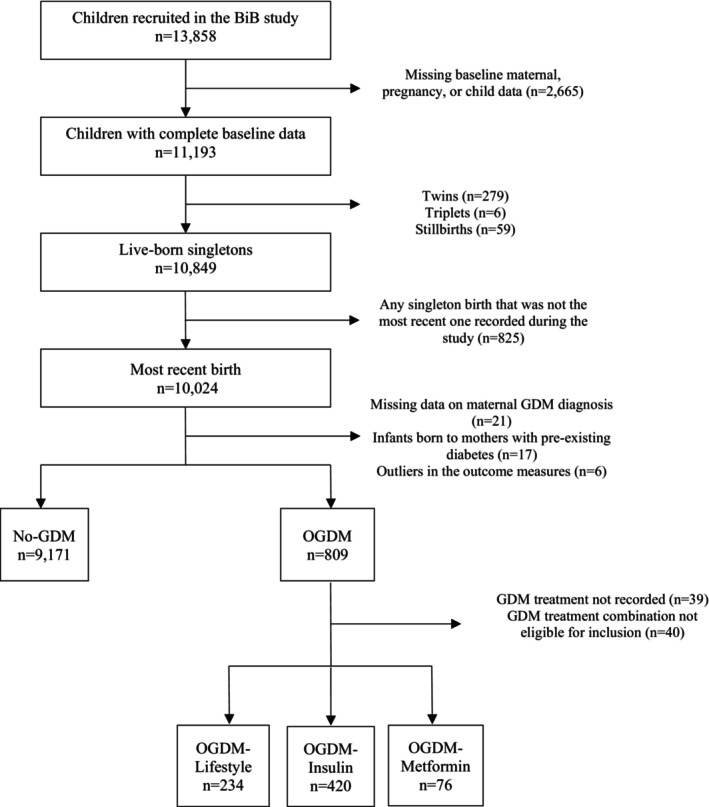
Flowchart of study participation.

Of the 13,858 children recruited to the BiB cohort, 10,849 were from live‐born singleton pregnancies with maternal, pregnancy and birth data (Figure 1). When mothers had more than one singleton pregnancies, any singleton birth that was not the most recent one recorded in the study was excluded (*n* = 825). We further excluded offspring with missing data on maternal treatment type or born to mothers with pre‐existing diabetes and GDM treatment combinations yielding insufficient numbers to conduct meaningful analyses. As shown in Figure 1, the final sample included 730 offspring born to mothers with GDM (OGDM) who received treatment and 9171 offspring born to mothers without GDM (No‐GDM).

### 2.3 Exposure

There were three main exposure groups: OGDM‐Metformin (*n* = 76), OGDM‐Insulin (*n* = 420) and No‐GDM (*n* = 9171). OGDM‐Metformin referred to OGDM exposed to lifestyle changes advice and metformin treatment and OGDM‐Insulin referred to OGDM exposed to lifestyle changes advice and insulin treatment. We also included OGDM‐Lifestyle (OGDM exposed to lifestyle changes advice alone, *n* = 234) for additional analyses (see statistical analysis section).

### 2.4 Outcomes

Three offspring growth outcomes were investigated: weight‐for‐age, height‐for‐age and BMI‐for‐age z‐scores from 0 to 60 months, according to the WHO Child Growth Standards.^13^ These measurements were available from maternal hospital notes, child health records, the BiB 1000 study (a subgroup of the BiB cohort),^14^ the National Child Measurement Programme (NCMP)^15^ and primary care data sets. Birthweight was measured with SECA® digital scales shortly after delivery. After birth, weight and height measurements were obtained by health visitors or researchers as part of routine clinical visits or for the BiB 1000 study.^11^, ^14^ BiB cohort children of school age (4–5 years old) also had growth measurements collected by nurses at school as part of the UK NCMP. The NCMP started in 2005, height and weight data were collected in primary school children to examine obesity prevalence and provide local authorities with evidence to inform decisions regarding services required to address childhood obesity.^15^

### 2.5 Covariables

In line with previous BiB evidence,^16^, ^17^ the following maternal characteristics were considered as covariables: body mass index (BMI), height, age at childbirth, parity, smoking during pregnancy, ethnicity, fasting and 2 h glucose concentrations at OGTT. Gestational age at delivery, route of birth and child sex were added to the model as competing exposures. Self‐reported age, parity (0, 1, 2 or 3+ children), smoking and ethnicity were obtained at recruitment from interviewer‐led questionnaires. Ethnicity (White British, Pakistani, Other) was reported in line with the Office of National Statistics guidelines.^18^ Maternal BMI at booking was calculated from weight measured at pregnancy booking (10–14 weeks of gestation) using SECA® digital scales and height at baseline (26–28 weeks of gestation) with Leicester Height measure. Fasting and 2‐h post‐load glucose concentrations at OGTT were measured using a glucose oxidase method. Gestational age at delivery and route of birth (vaginal or caesarean) were obtained from maternal health records.

### 2.6 Statistical analyses

All analyses were conducted using Stata/SE software (Stata/SE 15 for Windows; StataCorp).

#### 2.6.1 Primary analyses

We used multilevel linear spline models to estimate the weight, height and BMI z‐score trajectories of OGDM‐Metformin relative to OGDM‐Insulin (Aim 1) and OGDM‐Metformin and OGDM‐Insulin relative to No‐GDM (Aim 2). The process of knot selection for the models is described in the Supplementary Material. Knots placed at 1.6, 6 and 17 months were selected for the analysis.

For each outcome, we fitted three growth trajectory models: unadjusted, adjusted for maternal ethnicity and child sex and adjusted for all covariables. The models included a constant term (birthweight/height/BMI z‐score) and four linear spline terms (average change in weight/height/BMI z‐score between 0–1.6, 1.6–6, 6–17 and 17–60 months). Growth measurement occasion was at level 1 and offspring at level 2, under the assumption that data are missing at random.^19^ GDM treatment was added to the model and interaction terms were fitted between GDM treatment type and linear splines to evaluate differences in growth trajectories relative to a reference group (i.e. OGDM‐Insulin and No‐GDM) in each growth period. Measurement source was included as a fixed effect and the residuals were allowed to vary according to the measurement source. Measurements from the BiB 1000 study were categorised as research measurements while measurements from any other sources (hospital notes, health records, primary care and NCMP) were categorised as routine measurements. The full equation is provided in the Supplementary Material.

The normality of the level 1 residuals was assessed using normal probability plots (Q‐Q and P‐P plots) and histograms.

#### 2.6.2 Additional analysis

Using the model described in the primary analyses, we estimated the differences in growth trajectories between OGDM‐Lifestyle and No‐GDM. This was to determine whether the associations between pharmaceutical treatment exposure and offspring growth trajectories, relative to No‐GDM, were comparable to lifestyle changes advice exposure relative to No‐GDM.

## 3 RESULTS

Maternal and child baseline characteristics are shown in Table 1. Differences between groups have been described previously.^16^ Mean maternal age, BMI and glucose levels were the highest for OGDM‐Insulin and OGDM‐Metformin. There was a higher proportion of Pakistani women among the OGDM groups than there was among the No‐GDM group.

**TABLE 1:** Maternal and offspring characteristics.

	OGDM‐Metformin (*n* = 76)	OGDM‐ Insulin (*n* = 420)	OGDM‐Lifestyle (*n* = 234)	No‐GDM (*n* = 9171)	Missing *n* (%)
Maternal characteristics					
Age at childbirth (years), mean (SD)	31.1 (6.0)	32.0 (5.2)	30.0 (5.4)	27.9 (5.5)	0
Height (cm), mean (SD)	159.9 (5.8)	159.7 (6.5)	159.0 (6.4)	161.8 (6.4)	222 (2.2)
BMI at booking (kg/m^2^), mean (SD)	29.6 (6.2)	29.2 (6.3)	25.8 (5.0)	25.9 (5.6)	491 (5.0)
Ethnic group, *n* (%)					18 (0.2)
White British	15 (19.7)	112 (26.7)	44 (18.9)	3800 (41.5)	
Pakistani	54 (71.0)	238 (56.7)	142 (61.2)	3950 (43.1)	
Other	7 (9.2)	70 (16.7)	46 (19.8)	1405 (15.3)	
Parity, *n* (%)					362 (3.7)
0	21 (28.0)	120 (29.8)	85 (37.4)	3578 (40.5)	
1	20 (26.7)	96 (23.8)	49 (21.6)	2629 (29.8)	
2	21 (28.0)	74 (18.4)	40 (17.6)	1475 (16.7)	
3+	13 (17.3)	113 (28.0)	53 (23.3)	1152 (13.0)	
Smoking, *n* (%)					20 (0.2)
Yes	6 (7.9)	44 (10.5)	12 (5.1)	1602 (17.5)	
No	70 (92.1)	375 (89.5)	222 (94.9)	7550 (82.5)	
Gestational age at OGTT (weeks), mean (SD)	26.2 (1.8)	26.0 (2.3)	26.8 (2.5)	26.3 (1.9)	396 (4.0)
Fasting glucose concentrations at OGTT (mmol/L), mean (SD)	5.0 (0.7)	5.4 (1.0)	4.9 (0.7)	4.4 (0.4)	407 (4.1)
2‐h post‐load glucose concentrations at OGTT (mmol/L), mean (SD)	8.9 (1.2)	9.2 (1.7)	8.4 (1.2)	5.4 (1.0)	406 (4.1)
Gestational age at delivery (weeks), mean (SD)	38.3 (0.8)	38.1 (1.0)	39.1 (1.5)	39.7 (1.7)	0
Route of birth, *n* (%)					0
Vaginal	52 (68.4)	282 (67.1)	175 (74.8)	7225 (78.8)	
Caesarean	24 (31.6)	138 (32.9)	59 (25.2)	1946 (21.2)	
Offspring characteristics					
Child sex, *n* (%)					0
Girls	33 (43.4)	206 (49.0)	109 (46.6)	4459 (48.6)	
Boys	43 (56.6)	214 (50.9)	125 (53.4)	4712 (51.4)	

Abbreviations: BMI, body mass index; OGTT, oral glucose tolerance test.

There was a median of 6 (IQR 4–9) weight measurements and 4 (IQR 3–5) height and BMI measurements per child (Table S1). Assessment of model validity showed good model fit for all outcomes as suggested by the level 1 residuals and their SD (Table S2).

### 3.1 Primary analyses

Results of unadjusted models and models adjusted for maternal ethnicity and child sex are shown in Tables S3 and S4. Those were similar to the results of the fully adjusted models described in the sections below.

#### 3.1.1 OGDM‐Metformin versus OGDM‐Insulin

**FIGURE 2:**
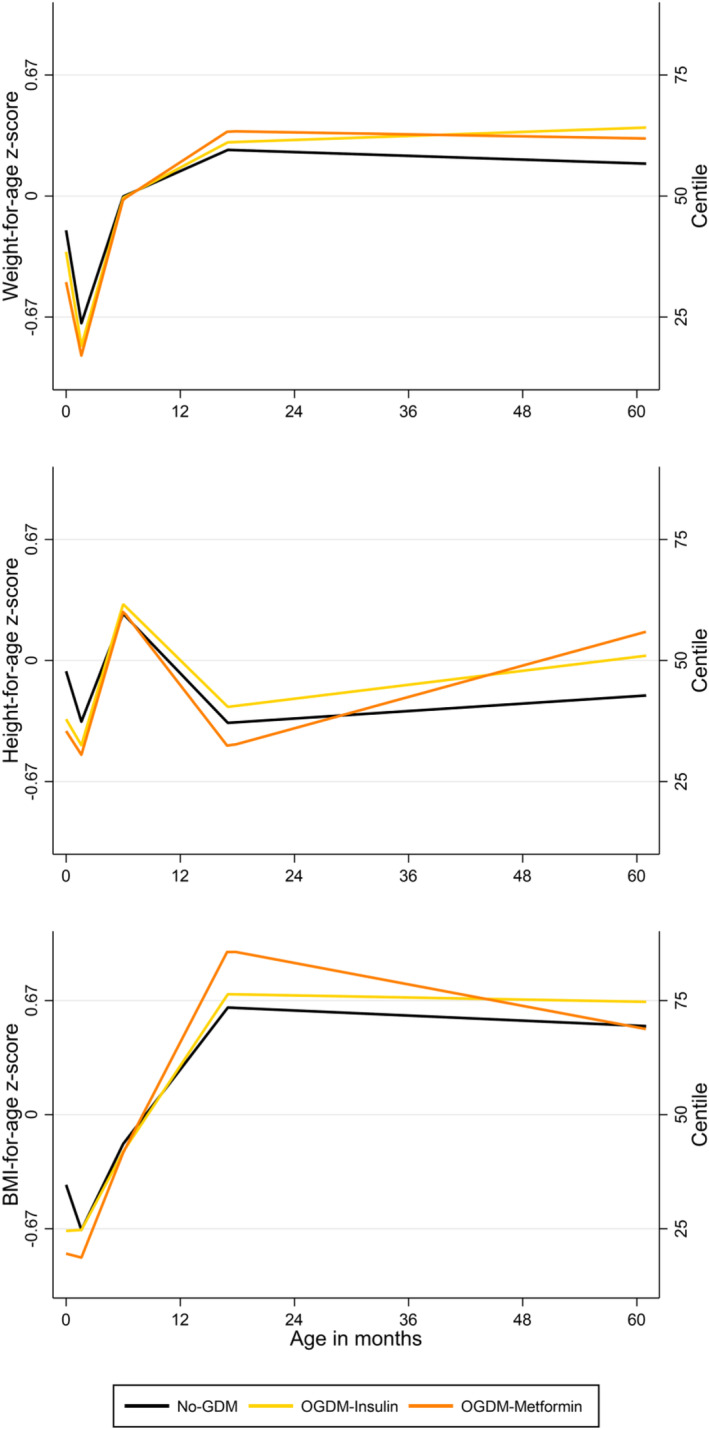
Weight, height and body mass index (BMI) z‐score trajectories of OGDM‐Metformin, OGDM‐Insulin and No‐GDM (fully adjusted models).

At birth, OGDM‐Metformin had comparable mean weight‐ (−0.17 [95% CI −0.40, 0.064]), height‐ (−0.06 [−0.5, 0.4]) and BMI‐for‐age (−0.13 [−0.56, 0.29]) z‐scores to OGDM‐Insulin. From 0 to 60 months, OGDM‐Insulin and OGDM‐Metformin followed similar weight and height growth trajectories. By 60 months, OGDM‐Metformin were predicted to have comparable mean BMI z‐score to OGDM‐Insulin (−0.16 [−0.55, 0.23]) (Table 2, Figure 2).

#### 3.1.2 OGDM‐Metformin versus No‐GDM

At birth, OGDM‐Metformin had lower mean weight (−0.29 [−0.50, −0.068]) and BMI (−0.40 [−0.81, 0.00036]) z‐scores than No‐GDM. There were no differences in growth trajectories between the two groups from 0 to 17 months. From 17 to 60 months, OGDM‐Metformin had faster changes in height z‐score (0.13 [0.026, 0.24]) while similar changes in weight were predicted during that period between the two groups. At 60 months of age, OGDM‐Metformin had comparable mean BMI z‐score to No‐GDM (−0.017 [−0.39, 0.35]) (Table 2, Figure 2).

#### 3.1.3 OGDM‐Insulin versus No‐GDM

OGDM‐Insulin had lower mean birthweight and height z‐scores than No‐GDM. OGDM‐Insulin had faster changes in weight and height z‐scores than No‐GDM, especially from 1.6 to 6 months (weight: 0.32 [0.021, 0.62], height: 0.50 [0.087, 0.91]). By 60 months of age, OGDM‐Insulin had comparable mean BMI z‐score to No‐GDM (0.14 [−0.019, 0.30]) (Table 2, Figure 2).

**TABLE 2:** Changes in weight‐, height‐ and BMI‐for‐age z‐scores from birth to 60 months in OGDM‐Metformin versus OGDM‐Insulin, OGDM‐Metformin versus No‐GDM and OGDM‐Insulin versus No‐GDM.

	OGDM‐Metformin vs. OGDM‐Insulin (ref)		OGDM‐Metformin vs. No‐GDM (ref)		OGDM‐Insulin vs. No‐GDM (ref)	
	Coefficients* (95% CI)	*p*	Coefficients* (95% CI)	*p*	Coefficients* (95% CI)	*p*
Weight‐for‐age z‐score						
Birth	−0.17 (−0.40, 0.064)	0.15	−0.29 (−0.50, −0.068)	0.010	−0.12 (−0.23, −0.0076)	0.036
0–1.6 months	0.85 (−0.90, 2.60)	0.34	0.80 (−0.81, 2.42)	0.33	−0.049 (−0.75, 0.66)	0.89
1.6–6 months	0.12 (−0.63, 0.88)	0.75	0.44 (−0.25, 1.14)	0.21	0.32 (0.021, 0.62)	0.036
6–17 months	0.076 (−0.24, 0.40)	0.64	0.13 (−0.17, 0.43)	0.39	0.054 (−0.071, 0.18)	0.40
17–60 months	−0.033 (−0.13, 0.063)	0.50	0.0099 (−0.081, 0.10)	0.83	0.043 (0.0074, 0.079)	0.018
60 months	−0.060 (−0.41, 0.29)	0.74	0.14 (−0.19, 0.47)	0.41	0.20 (0.050, 0.35)	0.009
Height‐for‐age z‐score						
Birth	−0.06 (−0.5, 0.4)	0.77	−0.33 (−0.75, 0.090)	0.12	−0.26 (−0.44, −0.087)	0.004
0–1.6 months	0.094 (−3.90, 4.09)	0.96	1.10 (−2.63, 4.84)	0.56	1.01 (−0.48, 2.49)	0.18
1.6–6 months	0.037 (−1.00, 1.07)	0.94	0.54 (−0.41, 1.49)	0.27	0.50 (0.087, 0.91)	0.018
6–17 months	−0.20 (−0.65, 0.26)	0.40	−0.16 (−0.58, 0.27)	0.47	0.039 (−0.13, 0.21)	0.66
17–60 months	0.096 (−0.017, 0.21)	0.095	0.13 (0.026, 0.24)	0.015	0.036 (−0.0037, 0.076)	0.076
60 months	0.13 (−0.21, 0.48)	0.45	0.35 (0.027, 0.68)	0.034	0.22 (0.075, 0.36)	0.003
BMI‐for‐age z‐score						
Birth	−0.13 (−0.56, 0.29)	0.54	−0.40 (−0.81, 0.00036)	0.050	−0.27 (−0.44, −0.10)	0.002
0–1.6 months	−0.22 (−4.21, 3.78)	0.91	1.80 (−1.93, 5.54)	0.34	2.02 (0.53, 3.51)	0.008
1.6–6 months	0.43 (−0.61, 1.47)	0.42	0.30 (−0.66, 1.26)	0.54	−0.12 (−0.54, 0.29)	0.55
6–17 months	0.28 (−0.22, 0.80)	0.27	0.42 (−0.057, 0.90)	0.084	0.13 (−0.057, 0.33)	0.17
17–60 months	−0.11 (−0.25, 0.025)	0.11	−0.096 (−0.23, 0.034)	0.15	0.017 (−0.033, 0.068)	0.50
60 months	−0.16 (−0.55, 0.23)	0.43	−0.017 (−0.39, 0.35)	0.93	0.14 (−0.019, 0.30)	0.083

* Estimates adjusted for source of growth measure, child sex, gestational age at delivery, route of birth, maternal BMI, maternal height, age at childbirth, maternal ethnicity, parity, fasting and 2‐h post‐load glucose concentrations at OGTT, smoking during pregnancy.

### 3.2 Additional analysis: OGDM‐Lifestyle versus No‐GDM

OGDM‐Lifestyle had lower mean birthweight (−0.23 [−0.36, −0.10]) and BMI (−0.25 [−0.45, −0.037]) z‐scores than No‐GDM. OGDM‐Lifestyle had comparable growth rates to No‐GDM from 0 to 60 months. By 60 months of age, OGDM‐Lifestyle had comparable mean BMI z‐score to No‐GDM (0.039 [−0.16, 0.24]) (Table S5).

## 4 DISCUSSION

This study is the first to examine the individual growth trajectories of OGDM exposed to maternal metformin or insulin treatment. It was demonstrated that OGDM‐Metformin had comparable birth size and weight, height and BMI z‐score trajectories to OGDM‐Insulin from 0 to 60 months. We have also shown that OGDM‐Metformin were born lighter than No‐GDM and faster changes in height z‐score between 17 and 60 months meant that by 60 months, OGDM‐Metformin had comparable BMI z‐score to No‐GDM. Lastly, OGDM‐Insulin were born lighter and shorter than No‐GDM and faster changes in weight and height z‐scores after birth for OGDM‐Insulin meant that by 60 months, they had comparable BMI z‐score to No‐GDM.

We have demonstrated that OGDM‐Metformin did not have faster changes in weight, height and BMI z‐scores than OGDM‐Insulin after birth and until 60 months of age. This is an important finding as it suggests that metformin use for GDM treatment in the BiB cohort was not associated with differences in offspring growth trajectories after birth in comparison to insulin use. In a recent meta‐analysis, there had been concerns regarding a potential accelerated post‐natal growth of OGDM‐Metformin compared to OGDM‐Insulin, as OGDM‐Metformin had lower birthweight but higher weight and BMI in childhood.^6^ However, the evidence in childhood was based on only three follow‐up studies and unlike our study, none of the studies included directly assessed the growth rates of OGDM‐Metformin and OGDM‐Insulin longitudinally, using a large number of repeated growth measurements across childhood. Further, the studies in the meta‐analysis^6^ showing higher weight and height at 18 months^7^ and weight and BMI by 9 years of age^8^ in OGDM‐Metformin than OGDM‐Insulin included in the metformin group both women treated with metformin alone and women requiring combined pharmaceutical treatment (metformin and insulin). However, previous evidence has suggested that women prescribed combined pharmaceutical treatment have higher baseline BMI or severity of hyperglycaemia than women prescribed metformin alone,^4^, ^20^ which may reflect a number of factors including differences in health behaviours between the two treatment groups. As parental health behaviours during pregnancy and after childbirth contribute to offspring post‐natal outcomes,^21^ we hypothesise that the inclusion of women prescribed combined pharmaceutical treatment in the metformin group in previous studies may have contributed to the observed higher childhood BMI in OGDM‐Metformin than OGDM‐Insulin. This is further supported by a recent study that has stratified children exposed to metformin alone and those exposed to insulin alone and shown that there was no increased risk in obesity associated with metformin exposure, relative to insulin, in children aged between 1 week and 11 years of age.^22^

We have shown that both OGDM‐Metformin and OGDM‐Insulin had comparable mean BMI z‐score to No‐GDM by 60 months of age. This is a key finding as it suggests that, regardless of the pharmaceutical treatment used for GDM in the BiB cohort, children exposed to treated maternal hyperglycaemia had comparable BMI z‐score by 60 months of age to children not exposed to maternal hyperglycaemia. Nevertheless, our results also highlighted faster changes in height z‐score in OGDM‐Metformin compared to No‐GDM and faster changes in height and weight z‐scores in OGDM‐Insulin compared to No‐GDM. Faster growth in OGDM exposed to treatment compared to No‐GDM has been described previously in weight and height from 12 to 14 months^23^ and in BMI from 6 months to 4–5 years^24^ and 2.5 years to 10 years old,^25^ but these studies did not stratify their results according to treatment type. Thus, what our study adds is the understanding that faster offspring growth was irrespective of metformin or insulin treatment. In OGDM‐Insulin, faster changes in weight z‐score could have important clinical implications if those persist after 60 months of age and translate into higher overweight and obesity risk later in childhood.^9^

It is hypothesised that GDM treatment interruption following childbirth may have contributed to the finding of faster growth in OGDM exposed to pharmaceutical treatment compared to No‐GDM as the effects of improved maternal glycaemic levels on offspring growth outcomes through treatment during pregnancy may be limited beyond birth. Clinical and socio‐demographic factors could have also contributed to the findings. We showed in previous work that maternal obesity and higher severity of maternal hyperglycaemia were both associated with higher odds of being prescribed GDM supplemental pharmaceutical treatment compared to lifestyle changes advice alone.^16^ Hence, adverse health risk factors in mothers receiving pharmaceutical treatment could have contributed to the finding that OGDM‐Metformin and OGDM‐Insulin, but not OGDM‐Lifestyle (as shown in additional analyses), had faster growth than No‐GDM. In addition, there was a higher proportion of Pakistani women among the OGDM groups than the No‐GDM group and it was shown in previous BiB research that children of Pakistani women were lighter at birth^26^ but taller by 4–5 years of age^17^ than those of White British women. Thus, maternal ethnicity could have also contributed to the differences observed in our study. However, even after adjustments for maternal characteristics, including maternal obesity, hyperglycaemia and ethnicity, the associations between exposure to pharmaceutical treatment and higher growth rates persisted. Thus, it is possible that other factors such as infant feeding methods (e.g. breastfeeding or formula feeding)^27^ and post‐natal parental health behaviours (e.g. smoking, physical activity levels)^28^ could have contributed to the associations observed, although we did not have the data to investigate this.

The main strength of this study is that the results were based on a large sample of OGDM and No‐GDM offspring born in a hospital where GDM was routinely tested and treated according to a standardised local protocol. Further, the timing of the BiB study meant that the data captured a key transition in local clinical practice with the introduction of metformin as a treatment for GDM. This allowed for the analysis of the individual growth trajectories of OGDM‐Metformin which had not been conducted previously. The granularity of the GDM treatment data meant that we were able to not only compare OGDM‐Metformin to OGDM‐Insulin but also OGDM‐Metformin, OGDM‐Insulin and OGDM‐Lifestyle to No‐GDM. Lastly, the analyses included an overall large number of repeated measurements and the multilevel linear spline design allowed for the assessment of changes in growth rates between key ages.

While our study provides a unique insight into the growth trajectories of OGDM‐Metformin and OGDM‐Insulin individually, we could not investigate the growth trajectories of offspring born to mothers who received both insulin and metformin treatment due to a small sample size and this may limit the applicability of our results to this clinical subgroup. The modified WHO criteria for GDM diagnosis^12^ used at the time of the BiB study differ from those that are currently used to diagnose GDM in England.^3^ While this may limit the applicability of our results to current clinical practice, our findings remain important by providing evidence of offspring growth outcomes when metformin was introduced to treat GDM in a UK hospital. Our study was also limited by the low number of birth length and BMI measurements as those were not routinely recorded. The estimates at birth predicted by the model should thus be interpreted with caution. No data on maternal breastfeeding habits and offspring lifestyle characteristics (e.g. physical activity levels, dietary habits) were available and thus it was not possible to control for their potential associations with offspring growth rates in our analyses.

To conclude, there was no evidence that metformin‐exposed offspring had different weight, height or BMI z‐score trajectories from 0 to 60 months than insulin‐exposed offspring. While both metformin‐ and insulin‐exposed offspring were predicted to grow faster than offspring not exposed to GDM after birth, there was no indication that this was associated with differences in BMI z‐score at 60 months of age.

## AUTHOR CONTRIBUTIONS

Gilberte Martine‐Edith wrote the manuscript and was responsible for the acquisition, analysis and interpretation of data. Emily S. Petherick and William Johnson revised the manuscript and contributed to the acquisition, analysis and interpretation of data. Gilberte Martine‐Edith, Emily S. Petherick and William Johnson are guarantors of this work and, as such, had full access to all the data in the study and take responsibility for the integrity of the data and the accuracy of the data analysis.

## FUNDING INFORMATION

This research was funded by Loughborough University and supported by the National Institute for Health Research (NIHR) Leicester Biomedical Research Centre. E.S.P. and W.J. acknowledge support from the National Institute for Health Research (NIHR) Leicester Biomedical Research Centre, which is a partnership between University Hospitals of Leicester NHS Trust, Loughborough University, and the University of Leicester. W.J. is supported by a UK Medical Research Council (MRC) New Investigator Research Grant (MR/P023347/1). Born in Bradford received funding from a Wellcome Trust infrastructure grant (WT101597MA) and the National Institute for Health and Care Research (NIHR) under its Collaboration for Applied Health Research and Care (CLAHRC) (IS‐CLA‐0113‐10020), now the Yorkshire and Humber Applied Research Collaboration (NIHR200166). The NIHR Clinical Research Network provided research delivery support for the BiB study. The views expressed in this publication are those of the authors and not necessarily those of the National Institute for Health and Care Research or the Department of Health and Social Care.

## CONFLICT OF INTEREST STATEMENT

None.

## Supporting information


Data S1.

## Data Availability

Scientists are encouraged and able to use BiB data. Data requests are made to the BiB executive using the form available from the study website http://www.borninbradford.nhs.uk. Guidance for researchers and collaborators, the study protocol and the data collection schedule are all available via the website. All requests are carefully considered and accepted where possible.
