# Supplementary material for: Associations between maternal gestational diabetes metformin or insulin treatment and offspring growth trajectories from birth to 60 months of age: Findings from the Born in Bradford (BiB) study
Source: Diabet Med. 2023 Aug 26;40(11):e15204. doi: 10.1111/dme.15204 (PMC10946820; doi:10.1111/dme.15204)
Supplement: Supplementary file 1 — Data S1. [file DME-40-0-s001.pdf]

Associations between maternal gestational diabetes metformin or insulin treatment and offspring growth trajectories from birth to 60 months of age: findings from the Born in Bradford (BiB) study

Gilberte Martine-Edith[a*], William Johnson[a], Emily S Petherick[a]

[a]*School of Sport, Exercise and Health Sciences, Loughborough University, Loughborough, UK*

Correspondence to: [*]Gilberte Martine-Edith, School of Sport, Exercise and Health Sciences, Loughborough University, Epinal Way, LE11 3TU, Loughborough, UK. E-mail: gilberte.martine_edith@kcl.ac.uk

Knot selection for the multilevel linear spline models

Knot selection for the linear spline models was conducted in a two-step process.

Firstly, restricted cubic spline models were fitted for each outcome over child age to allow for the identification of potential knot positions, as cubic polynomials allow for greater flexibility that better capture the shape of growth curves than linear polynomials[1].

Secondly, based on the cubic spline curves, a series of models with three knots were estimated for each outcome (knot at 0.9/1.1/1.4/1.6/1.8 months, with knot at 5/6/7/8/9/10 months and knot at 17/18/19/20/21/22 months). Knots placed at 1.6, 6 and 17 months were associated with the highest log likelihood and lowest Akaike and Bayesian Information Criterion and were selected for the analysis[2,3].

The equation of the final model for birth weight z-score (OGDM-Metformin vs OGDM-Insulin) was of the following form, for measurement occasion *i*, of an individual *j* (equivalent models were used for height and BMI):

$y_{ij} = \beta_{0j} +$

$\beta_{1j} \times (\text{Spline}_{1ij}) + \beta_{2j} \times (\text{Spline}_{2ij}) + \beta_{3j} \times (\text{Spline}_{3ij}) + \beta_{4j} \times (\text{Spline}_{4ij}) +$

$\beta_5 \times (\text{OGDM-Metformin}_j) +$

$\beta_6 \times (\text{OGDM-Metformin}_j \times \text{Spline}_{1ij}) + \beta_7 \times (\text{OGDM-Metformin}_j \times \text{Spline}_{2ij}) +$

$\beta_8 \times (\text{OGDM-Metformin}_j \times \text{Spline}_{3ij}) + \beta_9 \times (\text{OGDM-Metformin}_j \times \text{Spline}_{4ij}) +$

$\beta_{10} \times (\text{Covariable}_j) +$

$\beta_{11} \times (\text{Covariable}_j \times \text{Spline}_{1ij}) + \beta_{12} \times (\text{Covariable}_j \times \text{Spline}_{2ij}) +$

$\beta_{13} \times (\text{Covariable}_j \times \text{Spline}_{3ij}) + \beta_{14} \times (\text{Covariable}_j \times \text{Spline}_{4ij}) +$

$\beta_{15} \times (\text{Research measurement}_{ij}) +$

$e_{1ij} \times (\text{Research measurement}_{ij}) + e_{2ij} \times (\text{Routine measurement}_{ij})$

$$\beta_{0j} = \beta_0 + u_{0j}$$

$$\beta_{1j} = \beta_1 + u_{1j}$$

$$\beta_{2j} = \beta_2 + u_{2j}$$

$$\beta_{3j} = \beta_3 + u_{3j}$$

$$\beta_{4j} = \beta_4 + u_{4j}$$

$$\begin{bmatrix} u_{0j} \\ u_{1j} \\ u_{2j} \\ u_{3j} \\ u_{4j} \end{bmatrix} \sim N(0, \Omega_u) : \Omega_u = \begin{bmatrix} \sigma^2_{u_0} & & & & \\ \sigma_{u_{01}} & \sigma^2_{u_1} & & & \\ \sigma_{u_{02}} & \sigma^2_{u_{12}} & \sigma^2_{u_2} & & \\ \sigma_{u_{03}} & \sigma^2_{u_{13}} & \sigma^2_{u_{23}} & \sigma^2_{u_3} & \\ \sigma^2_{u_{04}} & \sigma^2_{u_{14}} & \sigma^2_{u_{24}} & \sigma^2_{u_{34}} & \sigma^2_{u_4} \end{bmatrix}$$

$$\begin{bmatrix} e_{1ij} \\ e_{2ij} \end{bmatrix} \sim N(0, \Omega_e) : \Omega_e = \begin{bmatrix} \sigma^2_{e1} & \\ 0 & \sigma^2_{e2} \end{bmatrix}$$

Where $\text{Spline}_{1\text{-}4ij}$ are the four linear splines, Covariable_j is a covariable that was considered in the model (offspring sex and maternal ethnicity were included as main effects but also their interaction with the 4 splines), $\text{Routine measurement}_{ij}$ is the reference level of the categorical variable measurement source and $\text{Research measurement}_{ij}$ is the second level of the categorical variable measurement source. $\beta_{0\text{-}15}$ are fixed effects, $u_{0\text{-}4j}$ are individual-level random effects, e_{1ij} the error term for research measurements and e_{2ij} the error term for routine measurements.

Table S1 Number of measurements per growth period

	Number of children with at least one measurement per growth period	Total number of measurements	Median (IQR) number of measurements per child
**Weight-for-age z-score**			
Overall	9,901	71,075	6 (4-9)
0 – 1.6 months	9,849	22,010	2 (2-2)
1.6 – 6 months	7,827	14,395	1 (1-2)
6 – 17 months	8,873	14,449	1 (1-2)
17 – 60 months	8,160	20,221	2 (1-3)
**Height-for-age z-score**			
Overall	9,755	44,039	4 (3-5)
0 – 1.6 months	7,987	8,392	1 (1-1)
1.6 – 6 months	7,381	8,234	1 (1-1)
6 – 17 months	8,687	11,242	1 (1-1)
17 – 60 months	7,531	16,171	2 (1-3)
**BMI-for-age z-score**			
Overall	9,748	43,145	4 (3-5)
0 – 1.6 months	7,885	8,281	1 (1-1)
1.6 – 6 months	7,227	8,023	1 (1-1)
6 – 17 months	8,645	11,109	1 (1-1)
17 – 60 months	7,463	15,732	2 (1-3)

Table S2 Actual measurements and predicted measurements by the model by GDM treatment group (fully adjusted model)

	**Weight Z-score**			**Height Z-score**			**BMI Z-score**		
	**Number of measurements**	**Mean actual measurement (SD)**	**Residual (SD)**	**Number of measurements**	**Mean actual measurement (SD)**	**Residual (SD)**	**Number of measurements**	**Mean actual measurement (SD)**	**Residual (SD)**
**0 – 1.6 months**									
No-GDM	17,966	-0.40 (1.14)	-0.0076 (0.22)	6,841	-0.23 (1.22)	-0.0030 (0.30)	6,752	-0.51 (1.03)	0.0022 (0.34)
OGDM-Lifestyle	470	-0.76 (1.14)	-0.013 (0.22)	179	-0.58 (1.16)	-0.0019 (0.27)	177	-0.72 (0.96)	0.0047 (0.32)
OGDM-Insulin	817	-0.67 (1.00)	-0.016 (0.24)	304	-0.72 (1.05)	-0.0084 (0.27)	302	-0.71 (0.97)	0.0058 (0.32)
OGDM-Metformin	147	-0.82 (1.01)	-0.017 (0.33)	53	-0.67 (1.06)	-0.0048 (0.33)	53	-0.88 (0.98)	0.0069 (0.34)
**1.6 – 6 months**									
No-GDM	11,827	-0.58 (1.12)	0.0043 (0.20)	6,702	-0.24 (1.24)	0.0016 (0.35)	6,534	-0.63 (1.04)	-0.0077 (0.36)
OGDM-Lifestyle	284	-0.93 (1.13)	0.021 (0.21)	183	-0.61 (1.32)	-0.010 (0.39)	177	-0.73 (1.01)	-0.0097 (0.35)
OGDM-Insulin	528	-0.71 (1.07)	0.012 (0.21)	313	-0.55 (1.15)	0.0027 (0.40)	303	-0.75 (1.00)	-0.015 (0.36)
OGDM-Metformin	82	-0.76 (1.04)	0.030 (0.20)	53	-0.65 (1.30)	-0.017 (0.53)	49	-0.97 (1.25)	-0.013 (0.44)
**6 – 17 months**									
No-GDM	11,810	0.035 (1.09)	0.012 (0.20)	9,197	0.22 (1.19)	0.0029 (0.40)	9,089	-0.046 (1.12)	0.0085 (0.38)
OGDM-Lifestyle	305	-0.26 (1.11)	0.0041 (0.21)	253	-0.068 (1.16)	0.0045 (0.40)	252	-0.25 (1.16)	0.018 (0.39)
OGDM-Insulin	573	-0.14 (1.24)	0.019 (0.22)	445	0.014 (1.28)	0.011 (0.39)	441	-0.17 (1.17)	0.016 (0.42)
OGDM-Metformin	87	-0.12 (1.18)	-0.0016 (0.18)	73	0.0068 (1.13)	0.023 (0.39)	72	-0.16 (1.17)	0.0040 (0.27)
**17 – 60 months**									
No-GDM	16,452	0.20 (1.09)	-0.0031 (0.21)	13,131	-0.12 (1.10)	-0.0015 (0.36)	12,792	0.46 (1.10)	-0.0031 (0.39)
OGDM-Lifestyle	463	0.0080 (1.22)	-0.0019 (0.20)	383	-0.29 (1.10)	0.0054 (0.36)	371	0.30 (1.22)	-0.011 (0.41)
OGDM-Insulin	834	0.13 (1.12)	-0.0057 (0.24)	652	-0.20 (1.15)	-0.0025 (0.36)	622	0.46 (1.15)	-0.0093 (0.40)
OGDM-Metformin	111	0.17 (1.20)	0.00093 (0.22)	86	-0.26 (1.20)	-0.0049 (0.36)	84	0.52 (1.15)	-0.00056 (0.33)

Table S3 Unadjusted estimates of the changes in weight, height and BMI z-scores from birth to five years

	**OGDM-Metformin vs OGDM-Insulin (Reference)**		**OGDM-Metformin vs No-GDM (Reference)**		**OGDM-Insulin vs No-GDM (Reference)**	
	Coefficients (95% CI)	p	Coefficients (95% CI)	p	Coefficients (95% CI)	p
**Weight-for-age z-score**						
Birth	-0.19 (-0.49, 0.097)	0.19	-0.47 (-0.74, -0.20)	0.001	-0.27 (-0.39, -0.15)	<0.001
0 – 1.6 months	0.88 (-0.88, 2.63)	0.33	0.94 (-0.68, 2.56)	0.26	0.063 (-0.64, 0.77)	0.86
1.6 – 6 months	0.18 (-0.58, 0.93)	0.64	0.50 (-0.19, 1.20)	0.16	0.33 (0.024, 0.63)	0.034
6 – 17 months	0.078 (-0.24, 0.40)	0.63	0.17 (-0.13, 0.47)	0.26	0.094 (-0.031, 0.22)	0.14
17 – 60 months	-0.024 (-0.12, 0.073)	0.62	0.019 (-0.072, 0.11)	0.69	0.043 (0.0073, 0.078)	0.018
60 months	-0.032 (-0.38, 0.32)	0.86	0.065 (-0.26, 0.39)	0.69	0.10 (-0.038, 0.23)	0.16
**Height-for-age z-score**						
Birth	-0.058 (-0.54, 0.42)	0.81	-0.56 (-1.02, -0.11)	0.014	-0.51 (-0.69, -0.33)	<0.001
0 – 1.6 months	0.18 (-3.89, 4.25)	0.93	1.34 (-2.46, 5.14)	0.49	1.16 (-0.34, 2.66)	0.13
1.6 – 6 months	0.15 (-0.89, 1.20)	0.77	0.74 (-0.22, 1.71)	0.13	0.59 (0.17, 1.01)	0.005
6 – 17 months	-0.18 (-0.64, 0.27)	0.42	-0.12 (-0.55, 0.30)	0.57	0.061 (-0.11, 0.23)	0.48
17 – 60 months	0.098 (-0.015, 0.21)	0.090	0.13 (0.026, 0.24)	0.015	0.035 (-0.0050, 0.075)	0.087
60 months	0.21 (-0.14, 0.56)	0.23	0.26 (-0.065, 0.58)	0.12	0.048 (-0.082, 0.18)	0.47
**BMI-for-age z-score**						
Birth	-0.17 (-0.62, 0.27)	0.45	-0.49 (-0.91, -0.074)	0.021	-0.32 (-0.49, -0.15)	<0.001
0 – 1.6 months	-0.13 (-4.17, 3.91)	0.95	1.88 (-1.89, 5.66)	0.33	2.01 (0.51, 3.51)	0.009
1.6 – 6 months	0.44 (-0.60, 1.48)	0.40	0.26 (-0.70, 1.23)	0.59	-0.18 (-0.59, 0.23)	0.39
6 – 17 months	0.29 (-0.22, 0.80)	0.26	0.45 (-0.025, 0.93)	0.063	0.16 (-0.033, 0.35)	0.10
17 – 60 months	-0.11 (-0.25, 0.027)	0.12	-0.092 (-0.22, 0.038)	0.17	0.020 (-0.031, 0.070)	0.45
60 months	-0.17 (-0.57, 0.23)	0.41	-0.067 (-0.44, 0.31)	0.73	0.10 (-0.051, 0.25)	0.19

Table S4 Changes in weight, height and BMI z-scores from birth to five years adjusted for maternal ethnicity and child sex

	**OGDM-Metformin vs OGDM-Insulin (Reference)**		**OGDM-Metformin vs No-GDM (Reference)**		**OGDM-Insulin vs No-GDM (Reference)**	
	Coefficients (95% CI)	p	Coefficients (95% CI)	p	Coefficients (95% CI)	p
**Weight-for-age Z-score**						
Birth	-0.16 (-0.45, 0.13)	0.28	-0.37 (-0.63, -0.10)	0.007	-0.21 (-0.32, -0.087)	0.001
0 – 1.6 months	0.85 (-0.90, 2.60)	0.34	0.81 (-0.80, 2.43)	0.32	-0.039 (-0.75, 0.67)	0.91
1.6 – 6 months	0.13 (-0.62, 0.89)	0.72	0.46 (-0.23, 1.16)	0.19	0.33 (0.026, 0.63)	0.033
6 – 17 months	0.074 (-0.25, 0.39)	0.65	0.13 (-0.17, 0.42)	0.41	0.053 (-0.072, 0.18)	0.40
17 – 60 months	-0.030 (-0.13, 0.066)	0.54	0.013 (-0.077, 0.10)	0.78	0.043 (0.0077, 0.079)	0.017
60 months	-0.039 (-0.39, 0.31)	0.83	0.076 (-0.25, 0.40)	0.65	0.11 (-0.021, 0.25)	0.097
**Height-for-age Z-score**						
Birth	-0.049 (-0.53, 0.43)	0.84	-0.51 (-0.96, -0.062)	0.026	-0.46 (-0.64, -0.28)	<0.001
0 – 1.6 months	0.26 (-3.80, 4.32)	0.90	1.26 (-2.54, 5.06)	0.51	1.00 (-0.50, 2.51)	0.19
1.6 – 6 months	0.059 (-0.98, 1.10)	0.91	0.56 (-0.40, 1.52)	0.26	0.50 (0.081, 0.91)	0.019
6 – 17 months	-0.20 (-0.66, 0.25)	0.38	-0.16 (-0.59, 0.26)	0.45	0.040 (-0.13, 0.21)	0.64
17 – 60 months	0.10 (-0.010, 0.21)	0.075	0.14 (0.031, 0.24)	0.011	0.035 (-0.0046, 0.074)	0.084
60 months	0.20 (-0.15, 0.54)	0.27	0.21 (-0.11, 0.54)	0.19	0.019 (-0.11, 0.15)	0.78
**BMI-for-age Z-score**						
Birth	-0.15 (-0.59, 0.30)	0.52	-0.40 (-0.82, 0.012)	0.057	-0.26 (-0.42, -0.093)	0.002
0 – 1.6 months	-0.023 (-4.06, 4.00)	0.99	1.92 (-1.85, 5.69)	0.32	1.94 (0.45, 3.44)	0.011
1.6 – 6 months	0.43 (-0.60, 1.47)	0.41	0.32 (-0.64, 1.29)	0.51	-0.11 (-0.52, 0.30)	0.60
6 – 17 months	0.28 (-0.23, 0.79)	0.28	0.41 (-0.063, 0.89)	0.089	0.13 (-0.061, 0.32)	0.18
17 – 60 months	-0.11 (-0.25, 0.024)	0.11	-0.095 (-0.22, 0.035)	0.15	0.019 (-0.031, 0.070)	0.45
60 months	-0.15 (-0.55, 0.25)	0.46	0.00098 (-0.37, 0.37)	0.99	0.15 (0.00070, 0.30)	0.049

Table S5 Changes in weight-, height- and BMI-for-age z-scores from 0-60 months of age in OGDM-Lifestyle relative to No-GDM

	**OGDM-Lifestyle vs No-GDM (Reference)**	
	**Coefficients* (95% CI)**	***p***
**Weight-for-age z-score**		
Birth	-0.23 (-0.36, -0.10)	<0.001
0 – 7 weeks	0.47 (-0.44, 1.39)	0.31
7 weeks – 6 months	-0.043 (-0.44, 0.35)	0.83
6 months – 17 months	0.068 (-0.094, 0.23)	0.41
17 months – 5 years	0.019 (-0.029, 0.067)	0.44
5 years	-0.055 (-0.24, 0.13)	0.57
**Height-for-age z-score**		
Birth	-0.15 (-0.37, 0.065)	0.17
0 – 7 weeks	-0.14 (-2.06, 1.78)	0.88
7 weeks – 6 months	-0.10 (-0.64, 0.43)	0.70
6 months – 17 months	0.14 (-0.075, 0.35)	0.20
17 months – 5 years	0.012 (-0.039, 0.064)	0.64
5 years	-0.039 (-0.22, 0.14)	0.67
**BMI-for-age z-score**		
Birth	-0.25 (-0.45, -0.037)	0.0021
0 – 7 weeks	1.74 (-0.18, 3.66)	0.075
7 weeks – 6 months	-0.081 (-0.61, 0.45)	0.77
6 months – 17 months	-0.055 (-0.30, 0.19)	0.66
17 months – 5 years	0.036 (-0.031. 0.10)	0.29
5 years	0.039 (-0.16, 0.24)	0.71

*Estimates adjusted for source of growth measure, child sex, gestational age at delivery, route of birth, maternal BMI, maternal height, age at childbirth, maternal ethnicity, parity, fasting and 2-hour post load glucose concentrations at OGTT, smoking during pregnancy

References

1. Grajeda LM, Ivanescu A, Saito M, et al. Modelling subject-specific childhood growth using linear mixed-effect models with cubic regression splines. *Emerg Themes Epidemiol*. Published online 2016. doi:10.1186/s12982-015-0038-3

2. Akaike H. A New Look at the Statistical Model Identification. *IEEE Trans Automat Contr*. Published online 1974. doi:10.1109/TAC.1974.1100705

3. Whittaker TA, Furlow CF. The comparison of model selection criteria when selecting among competing hierarchical linear models. *J Mod Appl Stat Methods*. Published online 2009. doi:10.22237/jmasm/1241136840
